# Homology modeling and docking studies of ENPP4: a BCG activated tumoricidal macrophage protein

**DOI:** 10.1186/s12944-016-0189-4

**Published:** 2016-01-28

**Authors:** Dongmei Yan, Weiwei Han, Zehua Dong, Qihui Liu, Zheng Jin, Dong Chu, Yuan Tian, Jinpei Zhang, Dandan Song, Dunhuang Wang, Xun Zhu

**Affiliations:** Department of Immunology, College of basic Medical sciences, Jilin University, Xinmin Street 126#, Changchun City, Jilin Province China; Key Laboratory for Molecular Enzymology and Engineering, Ministry of Education, Jilin University, Qianjin Street 2699#, Changchun City, Jilin Province China; Intensive Care Unit, The Affiliated Hospital of Qingdao University, Jiangsu Road 16#, Qingdao, China

**Keywords:** Macrophages, ENPP4 protein, Homology modeling, Molecular docking, Anti-tumor activity

## Abstract

**Background:**

The 3D structure and functions of ENPP4, a protein expressed on the surface of Bacillus Calmette–Guerin (BCG)-activated macrophages, are unknown. In this study, we analyzed the 3D structure of ENPP4 and determined its tumoricidal effects on MCA207 cells.

**Results:**

Homology modeling showed that Arg305, Tyr341, Asn291, and Asn295 are important residues in substrate, adenosine triphosphate (ATP), binding. A molecular dynamics study was also carried out to study the stability of ENPP4 (including zinc atoms) as well as its ligand–enzyme complex. BCG increased ENPP4 expression in macrophages, and specific blocking of ENPP4 in BCG-activated macrophages (BAMs) significantly reduced their cytotoxicity against MCA207 cells.

**Conclusions:**

These results indicate that zinc remains inside the ENPP4 protein, a BCG activated tumoricidal macrophage protein, throughout the simulation. Important information for the design of new inhibitors was obtained.

**Electronic supplementary material:**

The online version of this article (doi:10.1186/s12944-016-0189-4) contains supplementary material, which is available to authorized users.

## Background

Macrophages are the first line of defense of the human body against cancer development. Bacillus Calmette–Guerin (BCG) is a highly effective mediator that initiates the tumoricidal effect of macrophages through direct contact [1–5]. However, the mechanism of the antitumor effects of BCG remains unknown.

BCG can bind with TLR2 and TLR4 and upregulate certain proteins expressed on the macrophage surface or secreted to the microenvironment [6]. Activated macrophages can secrete large amounts of pro-inflammatory cytokines, such as tumor necrosis factor-α(TNF-α) and interlukine-12(IL-12), Nitric oxide(NO), reactive oxygen intermediates and highly express MHC-II and the costimulatory molecules CD80 and CD86 [7]. These molecules contribute to the tumoricidal effects of macrophages. Aside from these molecules [8], other membrane proteins involved in anti-tumor activities have been found to exist on the surface of BCG-activated macrophage (BAMs), as proven by the LS-LSM/MS technique.

Ectonucleotide pyrophosphatase–phosphodiesterases 4 (ENPP4) is an upregulated protein expressed on the BAM surface [2, 3]. However, its functions are incompletely understood. The nucleotide pyrophosphatase–phosphodiesterase or ectonucleotide pyrophosphatase–phosphodiesterase family includes eight members (NPP1–NPP8 or ENPP1–ENPP8) [9–11]. ENPP1–ENPP3 are composed of two N-terminal somatomedin B (SMB)-like domains (SMB1 and SMB2), a catalytic domain, and a nuclease-like domain (ENPP3 is monomeric). These phosphodiesterases are type-2 transmembrane metalloenzymes and have a short intracellular N-terminus, a single transmembrane region, and an extracellular domain that contains a catalytic site. By contrast, members ENPP4–ENPP7 consist of a single catalytic domain. Isoforms ENPP4 and ENPP5 are type-1 transmembrane proteins with a short intracellular C-terminus and a small extracellular region that contains only a phosphodiesterase motif.

The related physiological and pathological roles of ENPP family members, including their regulation of extracellular pyrophosphate levels, cell motility, migration, angiogenesis, and tumor cell invasion, have recently become the focus of intensive research [12–15]. The isozyme ENPP1 catalyzes nucleotides that generate pyrophosphate, which can prevent excessive bone calcification. The subtype ENPP2 (or autotaxin) hydrolyzes lysophosphatidylcholine into lysophosphatidic acid and is involved in vasculature and neural tube formation and lymphocyte migration [12]. The enzyme ENPP3 (CD203) is a basophile marker [13] with a glycosylated type II transmembrane structure. ENPP5 has not been widely studied, but its closely related molecule, ENPP2, has been widely implicated in neoplasia and extensively studied in efforts to develop small-molecule inhibitors [14]. In fact, ENPP2 has been identified as a novel angiogenesis-associated gene [15]. The isoform ENPP6 has lysophospholipase-C activity for choline-containing glycerophosphodiesters, and the isoform NPP7 has alkaline sphingomyelinase activity and catalyzes lysophosphatidylcholine. No study on the structure and functions of ENPP8 has yet been reported. Although some studies have reported the structure of ENPP4, its 3D structure and related functions are largely unknown.

The present study aims to analyze the 3D structure of ENPP4 on the BAM surface through molecular modeling and study its tissue distribution and tumoricidal effects through immunohistochemistry.

## Results

### Homology modeling

**Fig. 1:**
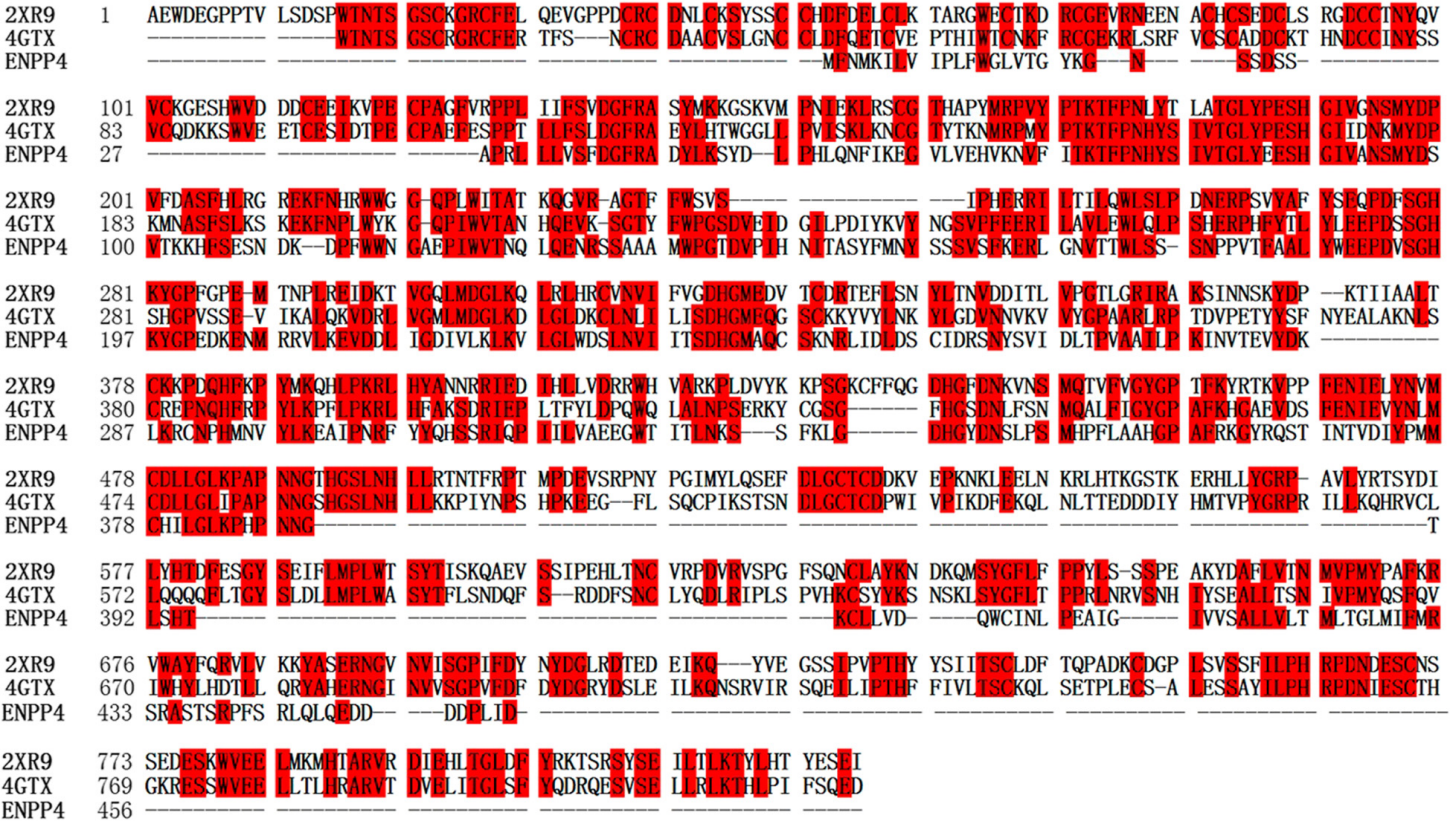
Sequence alignment of ENPP4 with other ENPP proteins. ENPP4 has 39 % sequence identity with PDB Id 4GTX (ENPP1) and 34 % sequence identity with PDB Id2XR9 (ENPP1)

**Fig. 2:**
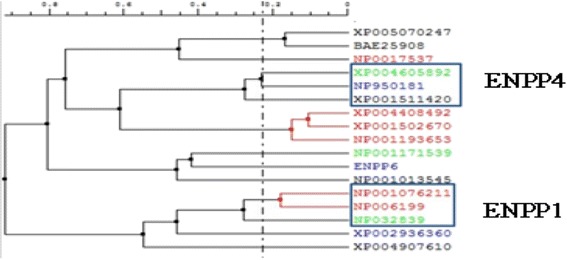
A phylogenetic tree of ENPP4 and related families

The search for the best template for modeling ENPP4 was carried out using PSI-BLAST against PDB. ENPP1 (PDB code4GTX) [10] showed the best sequence similarity with ENPP4 (39 %, Fig. 1). A phylogenetic tree of ENPP4 and its closest related sequences was constructed based on multiple sequence alignment (Fig. 2). This tree showed that ENPP4 has one clade, the ENPP4 subfamily. ENPP1 (PDB Id 4GTX) was selected as the template for ENPP4 modeling studies.

**Fig. 3:**
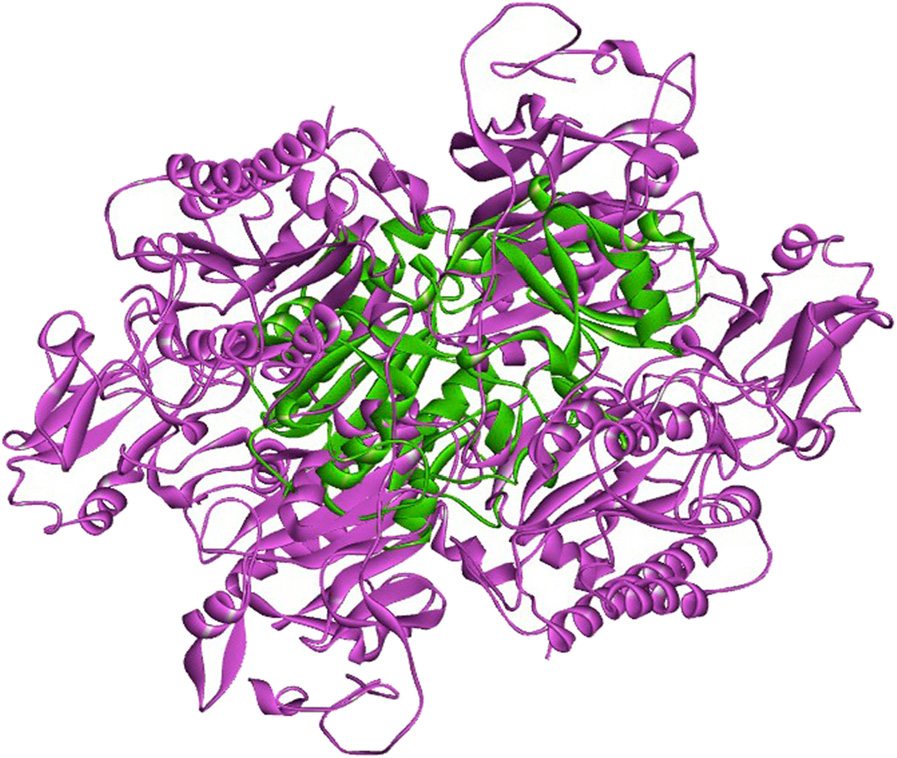
Overlay similarity between the ENPP4 (green) and the template ENPP1 (purple)

The initial model of ENPP4 was generated using a Swiss model. The initial model was refined by an energy minimization protocol in Gromacs 4.5.1. The final model was validated using four tools: Values of the root-mean square deviation (RMSD) from the initial structure were stable after approximately 2 ns (Additional file 1: Figure S1); such stabilization indicates that the trajectories have equilibrated. The final structure was further evaluated by Procheck. Ramachandran plot showed that the backbone dihedral angle distributions of all of the amino acid residues were 84.3 % in the core region, 13.4 % in the allowed region, and 1.3 % in the generously allowed region, as presented in Table 1. Meanwhile, the template ENPP1 (PDB Id 4GTX) presented 88.7 % in the core region and 11.3 % in the allowed region. These results indicate that the backbone dihedral angles of the ENPP4 model are reasonably accurate. The ERRAT score for ENPP4 was 86.36, whereas that for the template was 88.53 (Table 1); these scores are very similar. The final conformation with the lowest energy was chosen. Comparison of the model of the 3D structure of ENPP4 and the crystal structure of ENPP1 showed similar distributions of secondary structures (Fig. 3). The 3D structure supermposed between the ENPP1 and ENPP4 was 0.13 Å.

**Table 1:** The 3D models validation with Procheck, and Errat

Protein	Procheck	Errat
ENPP4	Ramachandran plot: 84.3 % core 13.4 % allow 1.3 % gener	86.36
ENPP1 (PDB Id 4GTX)	88.7 % core 11.3 % allow 0.0 % gener	88.53

All of these findings indicate that the 3D structure of ENPP4 obtained by MD simulation is reasonable and can be used for further investigations.

### Docking study

ENPP family proteins are membrane-bound glycoproteins. ENPP1 has been reported to cause human diseases characterized by ectopic calcification [16]. ENPP2 is a secreted lysophospholipase D that hydrolyzes lysophosphatidylcholine to produce lysophosphatidic acid (LPA), which, in turn, activates G protein-coupled receptors to evoke various cellular responses [17]. The five other mammalian ENPP proteins, ENPP3–ENPP7, have distinct substrate specificities and tissue distributions and thus participate in different biological processes. However, the 3D structure and biological functions of ENPP4 remain unknown.

ENPP1–ENPP3 are composed of two N-terminal SMB-like domains (SMB1 and SMB2), a catalytic domain, and a nuclease-like domain, whereas ENPP4–ENPP7 consist of a catalytic domain and lack the SMB-like and nuclease-like domains [16]. To determine the binding site between the protein and the substrate, the cavity volume was estimated by CASTp as a function of the radius of the probe sphere. A probe radius of 1.4 A outlined a cavity of 2237.4 Å^3^ for ENPP4 (Additional file 2: Figure S2A) and a cavity of 1177.7 Å^3^ for ENPP1 (Additional file 2: Figure S2B). The active pockets of ENPP4 and the template were similar. The active residues in ENPP1 were Asp358, His362, His517, Asp200, Thr238, Asp405, and His406. In ENPP4, Asp192, His196, His339 Asp37, Thr73, Asp240, and His241 were located in the catalytic domain. The reconstructed structure of ENPP4 reveals that the insertion loop (residues 139–158) participates in the formation of the substrate-binding pocket. In particular, M155 and S158 are located on the protein surface, whereas P140 and W138 are located at the bottom of the active pocket. The different binding pockets observed may influence the substrate specificities of the two proteins.

**Fig. 4:**
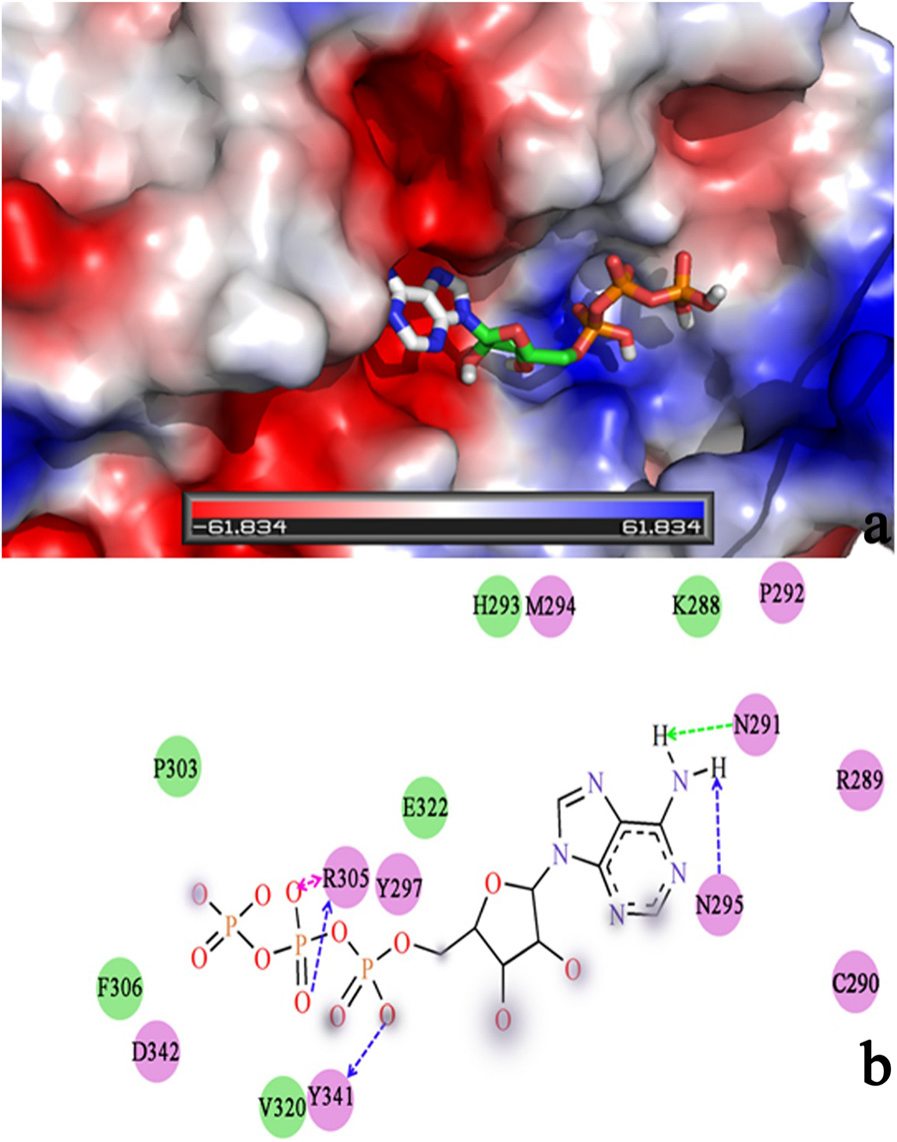
**a**: the substrate in the active site. **b**: ENPP4 -ATP interactions

The substrate, ATP, was docked in ENPP4. Figure 4a shows the binding pose of ATP in ENPP4. The substrate is located in the catalytic cleft and fits the binding pocket well. The interactions of ATP in the active cleft of ENPP4 are shown in Fig. 4b. Five hydrogen bonds were observed between ATP and ENPP4. Figure 4b shows that Arg305 forms two hydrogen bonds with ATP. The OH group of Tyr341 produces a hydrogen bond with ATP, and the adenosine group of ATP forms two hydrogen bonds with Asn291 and Asn295. Asp342, Tyr341, Arg305, Tyr297, Met294, Pro292, Asn291, Asn295, Arg289, and Cys290 show electrostatic interactions with ENPP4, whereas Pro303, Phe306, Val320, Glu322, His293, and Lys288 show van der Waals interactions with ENPP4. These results may be helpful in designing future ATP analogue inhibitors. The ligand was mainly stabilized by the hydrogen bonds determined during our MD simulation. Arg305, Tyr341, Asn291, and Asn295 allowed hydrogen-bonding with ENPP4. Among these residues, only Tyr431, which is located near the His339-coordinated Zn^2+^ in the ENPP4 family (Fig. 1).

### Construction of pET-28a–ENPP4 and expression of recombined ENPP4 protein and its polyantibody

**Fig. 5:**
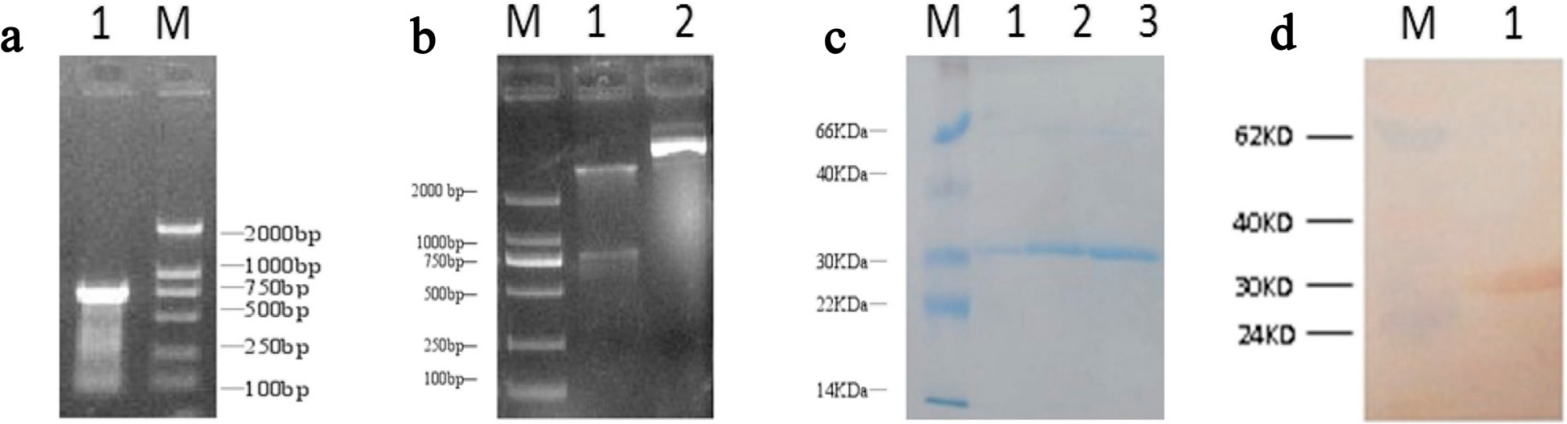
**a** Analysis of ENPP4 cDNA sequence amplified by RT-PCR. **b** Enzyme restriction assay with restriction enzymes EcoRI and XhoI of pET-28a-ENPP4 vector construction. Lanes 1 shows the digested plasmids with its expected sizes released from constructed DNA-vectors. Lanes 2 shows the non-digested plasmids. **c** SDS gel electrophoretic patterns of recombinant ENPP4 after purification. Lanes 1–3: different concentration of ENPP4 protein stained by Coomassie blue. **d** PVDF membrane of western blotting assay of ENPP4 purified protein (32KD)

The DNA fragment with an expected size of 750 bp was obtained by PCR and enzyme digestion with EcoRI and XhoI, which demonstrates that the correct pET-28a–ENPP4 plasmids were constructed (Fig. 5a and b). After the pET-28a–ENPP4 recombinant plasmid was transformed in *E. coli* Rosetta (DE3), the expression of ENPP4 was detected and shown in Fig. 5c and d, (32 KD MW protein). A large amount of recombinant ENPP4 protein was obtained in insoluble form. The resulting protein showed a purity of over 90 %, as determined by SDS-PAGE (Fig. 5c). Polyclonal antibodies were produced in rabbits. Figure 5d shows the high specificity of the anti-ENPP4 polyclonal antibodies for binding to ENPP4, as determined by Western blot detection.

### Expression of ENPP4 in tissues

**Fig. 6:**
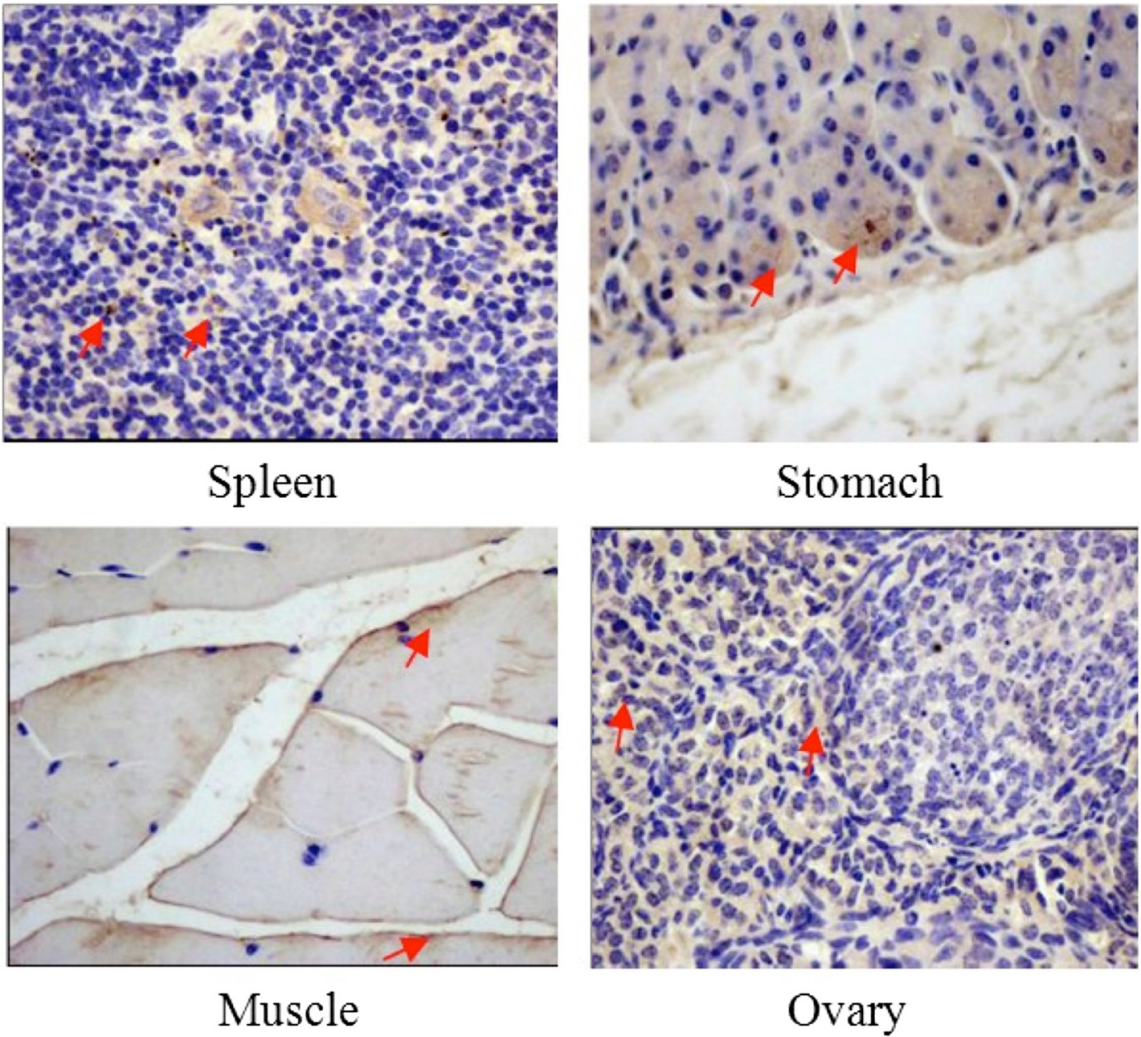
The expression of ENPP4 in different tissues, red arrow show abundant expression. Scoring was completed by a specialist pathologist and a scientist who were blinded to the pathologic information (× 400)

The expression of ENPP4 was detected in 12 tissue samples from a normal female C57BL/6 mouse. ENPP4 was abundantly expressed in the spleen, stomach, and ovary (Fig. 6). No expression was observed in the brain, lung, kidney, thymus, liver, heart, uterus, and intestine. This result indicates that ENPP4 is involved in biological pathways related to immunity and reproduction.

### Tumoricidal activity of ENPP4 in BAMs

**Fig. 7:**
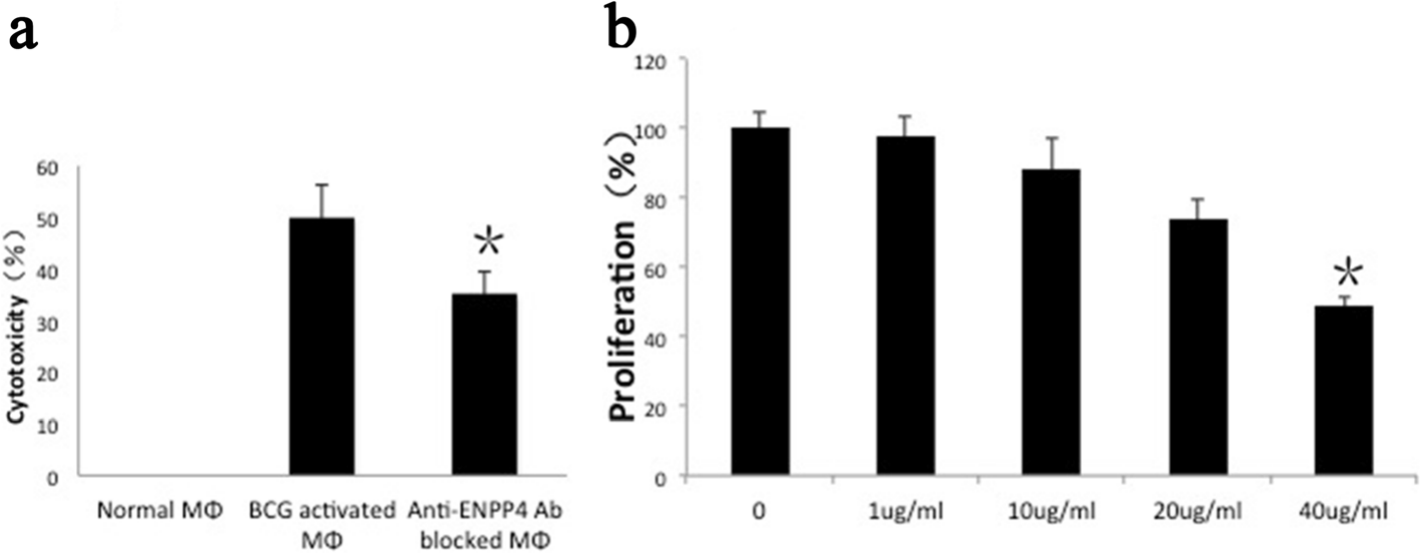
ENPP4 has tumoricidal activity against MCA207 cells. **a** Antibodies against ENPP4 influenced the tumoricidal activity of macrophages. Negative control cells exhibited no cytotoxic activity, whereas BCG-activated macrophages exhibited a cytotoxicity of 50 %. Blocking ENPP4 on BCG-activated macrophages decreased the cytotoxicity to 35.1 %. *, *P* < 0.05 compared with positive control. **b** ENPP4 protein inhibited the proliferation of MCA207 cells in a dose-dependent manner. *, *P* < 0.05 compared with negative control

To study the contact-dependent tumoricidal activity of ENPP4, cytotoxicity assays were carried out using paraformaldehyde-fixed macrophages. BAMs showed prominent cytotoxicity against MCA207 cells and this cytotoxic activities may be downregulated by blocking ENPP4 (Fig. 7a). The negative control did not exhibit cytotoxic effects. These results demonstrate that ENPP4 may be an essential functional molecule in the BAM-mediated killing of MCA207 cells. Furthermore, cytotoxicity experiment results showed that ENPP4 protein exerts direct tumoricidal activities against MCA207 cells (Fig. 7b).

## Discussion

MD simulation was employed to assess the stability of the homology model and the ligand–enzyme complex. In the homology model, we focused on the catalytic site, particularly on the amino acids that coordinate Zn^2+^ and the interactions between the metal and the enzyme. MD simulation was used to analyze the ENPP4–ATP complex and identify the possible bioactive conformation of the molecule. The conformation of ATP obtained from molecular docking and the ENPP4 model were used as the starting point for the simulation. The ligand was mainly stabilized by the hydrogen bonds determined during our MD simulation. Among Arg305, Tyr341, Asn291, and Asn295, only Tyr431 was conserved in the ENPP4 family. These observations suggest that this specific residue is necessary for the molecular evolution of the ENPP family. The plot shows the distance between the H in the oxygen of Tyr341 and ATP. Interestingly, the stable formation of a hydrogen bond between the ligand and Asp141 was observed during the 10 ns MD simulation. This finding suggests that the residue of the catalytic site can stabilize interactions between OH and ATP; however, other studies featuring longer simulations are necessary to support this idea.

*Mycobacterium bovis* BCG is the most widely used vaccine in the world. BCG generates a local immunological reaction that activates immune cells, including polymorphonuclear and mononuclear cells, in bladder tumors after BCG therapy [12]. The ENPP family has been reported to be involved in various pathologies, including tumor progression and inflammation. ENPP2 is a secreted lysophospholipase D that generates the lipid mediator LPA, a mitogen, and a known chemoattractant for many cell types [18]. We have certified that the expression of ENPP4 is upregulated by BCG (Additonal file 3: Figure S3). Blocking ENPP4 on BAM significantly downregulates the anti-tumor activity of the cell, which demonstrates that ENPP4 has potential tumoricidal activity.

ENPP4 showed a catalytic domain in Asp192, His196, His339 Asp37, Thr73, Asp240, and His241, which suggests that the ENPP4 may affect some receptor such as ATP receptor or insulin receptor on the surface of tumor cells to reduce their proliferation by indirectly or directly contact, receptively [19, 20]. On the one hand, ENPP4 may catalize the extracellular ATP released from tumor cells and reduce the binding between ATP and ATP receptor [19, 21], on the other hand, ENPP4 may contact the insulin receptor and inhibit the insulin receptor activity [20, 22].

Altergether, ENPP4 may thus be targeted as a therapeutic molecule for treating tumors. To explore the therapeutic potential of such a strategy, more detailed knowledge of the functions of ENPP4 and its ligand in tumor cells is needed.

## Conclusion

Our findings provide novel insights into the structure of ENPP4 and help researchers better understand its diverse cellular functions.

## Methods

All experiments conform to Jilin University guidelines on the ethical use of animals and were approved by the Institutional Animal Care and Use Committee. The mice used were C57BL/6 (wild-type, WT).

### Homology modeling

The amino acid sequence of the target protein, ENPP4, was obtained from the National Center for Biotechnology Information NCBI (http://www.ncbi.nlm.nih.gov/) (GenBank: NP_950181; template protein: ENPP1 (PDB Id 4GTX) [16]. The BLAST search algorithm was used for an online search (http://www.ncbi.nlm.nih.gov). A modeler module was employed to build the 3D structure of the protein. Modeling was then carried out using Gromacs 4.5.1 software [23] with the Gromos53a6 all-atom force field. The temperatures were kept constant at T = 25 °C by coupling to a Berendsen thermostat with a coupling time of T = 0.1 ps. The protein was solvated using a box of TIP3P [24] water molecules extending at least 10 Å away from the boundary of any protein atom with an integration step of 2 fs. Non-bonded interactions were calculated using a cutoff of 10 Å. Long-range electrostatic interactions were calculated by Particle–Mesh Ewald summation with a grid spacing of 1.2 Å and cubic interpolation. After 1000 steps of steepest descent energy minimization, the solvent and ions were equilibrated by 0.5 ns molecular dynamics (MD) simulation, with the heavy protein atoms subjected to harmonic constraints under a force constant of k = 1000 kcal/(mol^−1^ · nm^−2^). Finally, a production run was carried out for 10 ns, and the coordinates of all atoms at each picosecond were stored for further analysis. In our studies, CASTp (http://cast.engr.uic.edu/cast/) [25] was used to identify all of the cavities associated with the model and the template and measure their volumes. The results obtained were further used for protein-ligand docking investigations.

### Docking study

The software AutoDock Vina [26, 27] was applied for docking studies. The 3D structures of the substrate were downloaded from the Chemspider database. The target used in our study was the 3D structure of ENPP4. The grid size for docking measured 36 × 36 × 36 Å^3^.

### Cloning of ENPP4 gene in the expression vector pET-28a

To obtain a mouse peritoneal macrophage cell preparation, female C57BL/6 mice, 10 week of age, were immunized intraperitoneally (i.p.) with 4 mg of BCG (Chengdu Institute of Biological Products) three times on days 2, 10, and 12. Three days after the last i.p. injection, the mice were sacrificed and peritoneal cells were collected. Adherent macrophages were collected to prepare the total RNA and cDNA of ENPP4 by RT-PCR [2, 3]. Briefly, PCR reactions were performed using self-designed primer pairs: EF (5’-CGGGAATTCTCAGCACCTCGGTTACTT-3’) and ER (5’-AAACTCGAGAAGAATCGCAGCCACAGG-3’). All amplification reactions consisted of an initial denaturation step at 94 °C for 5 min, 30 cycles of 94 °C denaturation for 1 min, 57 °C annealing for 1 min, and 72 °C extension for 2 min, followed by a final extension at 72 °C for 10 min using 2 units of Taq DNA polymerase. The cDNA of ENPP4 was then cloned to pET-28a to obtain the pET-28a–ENPP4 plasmid.

### Expression and purification of recombinant ENPP4 protein in Escherichia coli

To determine the expression of the recombinant ENPP4 protein, *Escherichia coli* Rosetta (DE3) (Invitrogen) transformed with pET-28a–ENPP4 plasmid DNA was inoculated in a tube containing 10 mL of LB medium supplemented with kanamycin and chloromycetin. The tube was allowed to culture overnight at 37 °C in a shaking incubator (180 rpm) until the culture reached an OD of 0.6 when read at a wavelength of 600 nm. Protein expression was induced by the addition of 0.2, 0.4, 0.6, and 0.8 mM isopropyl βD-1-thiogalactopyranoside. After 6 h of induction at 37 °C, the cells were collected by centrifugation for sodium dodecyl sulfate-polyacrylamide gel electrophoresis (SDS-PAGE).

To obtain the recombinant ENPP4 protein, bacterial cells were harvested by centrifugation at 4 °C and resuspended in PBS buffer. The bacterial pellets were then mixed with 10 mmol/L MgSO4, 0.01 mg/mL DNaseI, and 0.1 mg/mL lysis enzyme and left at 4 °C for 20 min. After centrifugation, the bacterial pellets were loaded with ice-cold lysis buffer (50 mmol/L Tris-Cl, 0.1 mol/L NaCl, 5 mmol/L EDTA, 0.1 % NaN_3_, 0.5 % Triton X-100, pH 6.8) and sonicated at 1000 W for 5 s with 10 s intervals for 160 cycles. The supernatant was discarded, and the pellet (inclusion bodies) containing the recombinant ENPP4 protein was subjected to dilute renaturation and dialysis renaturation and concentration. The purity of the extracted protein was detected by SDS-PAGE.

### Preparation of polyclonal antibodies and Western blot analysis

Purified recombitant protein was used as the immunogen. Approximately 100 μg of the fusion protein emulsified in complete Freund’s adjuvant was administered during initial inoculation, and 50 μg of fusion protein emulsified in incomplete Freund’s adjuvant was given after 2 weeks. This procedure was repeated 5 times at 1 week intervals. Final rabbit serum was harvested after immunization.

ENPP4 peptides were separated by SDS-PAGE and electrotransferred onto the PVDF membrane. The membrane was blocked with 3 % bovine serum albumin in PBS for 2 h at room temperature. Subsequently, anti-ENPP4 antibodies were added at a dilution of 1:1000 and incubated at room temperature for 1 h. After washing thrice for 15 min, IgG goat-anti-rabbit-HRP was added and incubated for 1 h at room temperature. After a final washing, the resulting signals were detected using DAB (3,3-diaminobenzidine tetrahydrochloride).

### Immunohistochemistry for ENPP4 detection in mouse tissues

Various mouse tissues (including heart, liver, kidney, spleen, muscle, lung, intestine, uterus, and ovary) were formalin-fixed at 4 °C overnight and then embedded in paraffin and sectioned for histology. Immunohistochemical staining of serial sections was performed to detect the expression and distribution of ENPP4. All samples were incubated in 0.3 % H_2_O_2_ for 10 min at room temperature to inactivate endogenous peroxidase. The paraffinized sections were then incubated with anti-ENPP4 primary antibody for 1 h at room temperature. After washing, the sections were incubated with peroxidase-conjugated goat anti rabbit IgG for 40 min. After a final washing, the resulting signals were detected using DAB and photographed with a microscope.

### Tumoricidal effect of ENPP4 on MCA207 cells

BAM-mediated tumor cytotoxicity was determined with and without anti-ENPP4 antibodies. Peritoneal macrophages were harvested from mice and fixed with 1 % paraformaldehyde for 30 min at room temperature. Afterward, 6 × 10^5^ cells were incubated for 2 h with 5 μL of anti-ENPP4 serum (previously prepared by our research group) and 5 μL of control serum (from preimmunized rabbit). The blocked cells were then washed twice with RPMI 1640 medium, seeded into a 96-well flat-bottom plate containing 1 × 10^4^ MCA207 target cells/well, and incubated for 48 h. The number of viable tumor cells was determined by MTT (5 mg/mL, Sigma) assay. The absorbance at 570 nm was recorded directly using a microplate reader (Model 550, Bio-RAD).

Purified ENPP4 protein (prepared by our team) was added to MCA207 (1 × 10^4^ cells, cultured in 96-well plates) at concentrations of 1, 10, 20, and 40 μg/mL, and the cells were incubated for 48 h at 37 °C under a 5 % CO_2_ atmosphere. The proliferation levels of MCA207 were detected by MTT assay.

### Statistical analysis

Each experiment was repeated at least three times. Data were expressed as mean ± SEM and analyzed by one-way analysis of variance. *P* values < 0.05 (95 % confidence level) were considered statistically significant.

## Additional files

Additional file 1: Figure S1. The RMSD of ENPP4 during the 10 ns MD simulation. (JPG 172 kb) Additional file 2: Figure S2 a: The binding pocked of ENPP4 (calculated by CASTp, 2237.4 Å3); b: The binding pocket of ENPP1 (PDB Id 4GTX) (calculated by CASTp,. 1177.7 Å3). (JPG 223 kb) Additional file 3: Figure S3. BCG increased the expression of ENPP4 in the macrophages. (JPG 22 kb)
