# Supplementary figures and images for: Homology modeling and docking studies of ENPP4: a BCG activated tumoricidal macrophage protein
Source: Lipids Health Dis. 2016 Jan 28;15:19. doi: 10.1186/s12944-016-0189-4 (PMC4730737; doi:10.1186/s12944-016-0189-4)

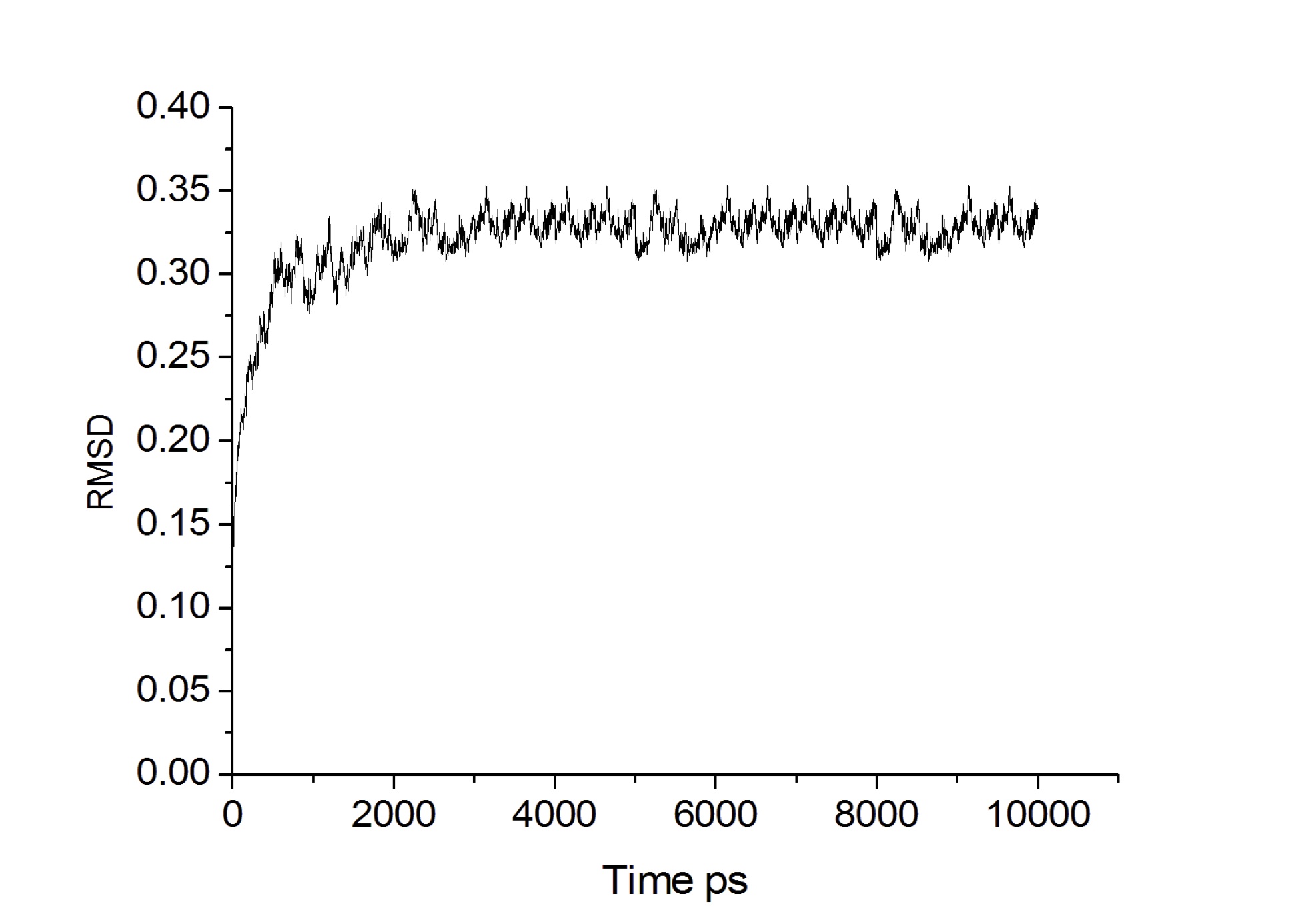

Supplement: Additional file 1: Figure S1. — The RMSD of ENPP4 during the 10 ns MD simulation. (JPG 172 kb) [file 12944_2016_189_MOESM1_ESM.jpg]

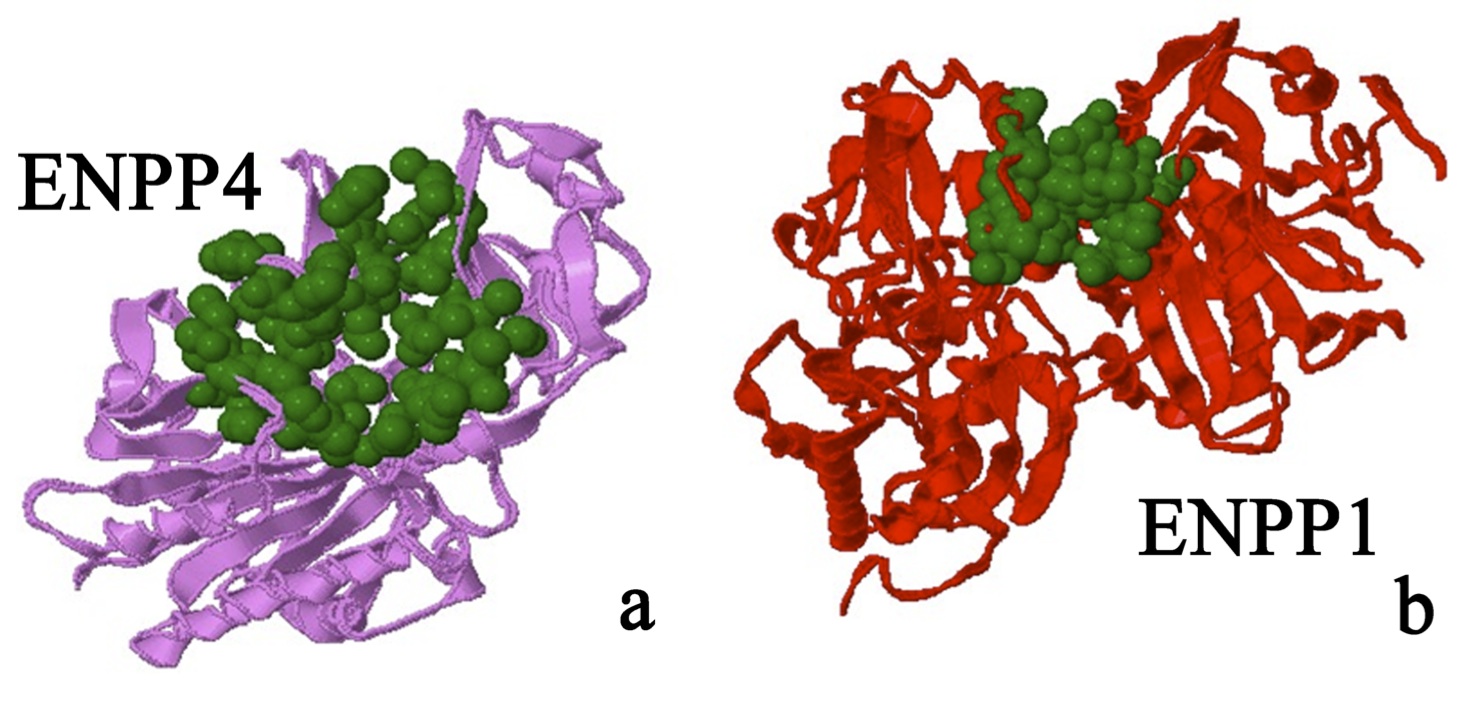

Supplement: Additional file 2: Figure S2 — a: The binding pocked of ENPP4 (calculated by CASTp, 2237.4 Å3); b: The binding pocket of ENPP1 (PDB Id 4GTX) (calculated by CASTp,. 1177.7 Å3). (JPG 223 kb) [file 12944_2016_189_MOESM2_ESM.jpg]

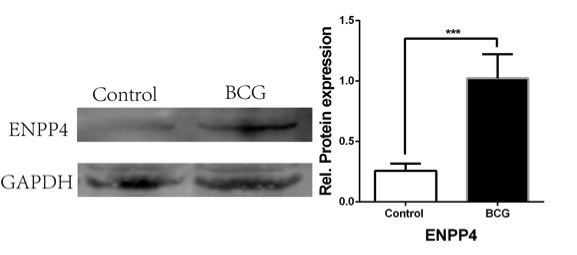

Supplement: Additional file 3: Figure S3. — BCG increased the expression of ENPP4 in the macrophages. (JPG 22 kb) [file 12944_2016_189_MOESM3_ESM.jpg]
